# MS in self-identified Hispanic/Latino individuals living in the US

**DOI:** 10.1177/2055217317725103

**Published:** 2017-09-25

**Authors:** Lilyana Amezcua, Jorge R Oksenberg, Jacob L McCauley

**Affiliations:** Department of Neurology, University of Southern California, Keck School of Medicine, USA; Department of Neurology, University of San Francisco School of Medicine, USA; Dr. John T. Macdonald Department of Human Genetics, Miller School of Medicine, University of Miami, USA; John P. Hussman Institute of Human Genomics, Miller School of Medicine, University of Miami, USA

**Keywords:** Multiple sclerosis, Latino/Hispanic, epidemiology, clinical presentation

## Abstract

Self-identified Hispanic/Latino individuals living with multiple sclerosis (MS) in the continental United States (US) are a diverse group that represents different cultural and ancestral backgrounds. A marked variability in the way MS affects various subgroups of Hispanics in the US has been observed. We reviewed and synthesized available data about MS in Hispanics in the US. There are likely a host of multifactorial elements contributing to these observations that could be explained by genetic, environmental, and social underpinnings. Barriers to adequate MS care in Hispanics are likely to include delivery of culturally competent care and social and economic disadvantages. Considerable efforts, including the formation of a national consortium known as the Alliance for Research in Hispanic Multiple Sclerosis (ARHMS), are underway to help further explore these various factors.

## Introduction

Multiple sclerosis (MS) is an immune-mediated, progressive, demyelinating, and degenerative disease. Well-documented differences in disease prevalence, age of onset, central nervous system site of injury, and progression have been observed across ancestral groups and are thought to be in part, the result of a complex interaction between genetic risk factors, lifestyle, and environmental exposures. In the United States (US), it is estimated that the number of Hispanics with MS will increase, given the estimates that the proportion of Hispanics in the US population will rise from 14% in 2005 to 29% by 2050 (web 1). Despite these numbers, Hispanics with MS in the US remain an understudied population.^1^

We review the currently available literature regarding this population in the US. In general, studies of self-identified Hispanics living in the US with MS have focused almost exclusively on describing clinical presentation, which appears to show a marked variability among different Hispanic subgroups.

## Methods

We identified all new epidemiological data regarding MS in Hispanics living in the continental US who were born in the US or arrived as Latin American immigrants. This search included original peer-reviewed studies published in English between January 2000 and December 2016. The search terms “multiple sclerosis” and “Hispanic” were entered in the MEDLINE/PubMed database. In addition, search terms such as “American,” “immigrant,” “migrant,” and/or “place of birth,” were also added, yielding 19 studies.

## Definitions

### Hispanic/Latino

The terms “Hispanic” or “Latino” are often used interchangeably to describe the ethnic group of individuals, living in the US, who themselves or their ancestors originate from Spain or from Latin American countries and/or those individuals who speak the Spanish language (web 1; Table 1). The terms “Hispanic” and “Latino” are not the same (Table 1). “Hispanic” historically refers to people born in regions of the Americas conquered by Spaniards and for whom Spanish is the primary language, whereas “Latino” is more inclusive, referring to people with ancestral and cultural ties to Latin America (which can include Brazil, where Portuguese is spoken) and within the bounds of that region (Table 1). Noteworthy, in 1997 the US government, regarding the ethnonym of this collective group of individuals, replaced the single term “Hispanic” with “Hispanic or Latino” and in practice, when referring to residents of the US who have a Latin American origin, the terms “Hispanic” and “Latino” are used interchangeably, with “Hispanic” being used more commonly. Throughout the remainder of this article we will generally use the term “Hispanic” to refer to this group.

**Table 1.** Definition of Hispanic/Latino in the United States (US).

Ethnicity term	Source	Race	Definition
“Hispanic or Latino”	NIH	Any race or combination of races: White/Caucasian, Black/African American, Asian, Native American, Native Hawaiian/Other Pacific Islander American, or two or more races.	A person of Cuban, Mexican, Puerto Rican, South or Central American, or other Spanish culture or origin, regardless of race.
“Hispanic or Latino”	US Census	Any race or combination of races: White/Caucasian, Black/African American, Asian, Native American, Native Hawaiian/Other Pacific Islander American, or two or more races.	A person of Cuban, Mexican, Puerto Rican, South or Central American, or other Spanish culture or origin, regardless of race.
Latino or Latino Americano	Non- governmental	Any race.	Term often used to refer to people with cultural ties to Latin America and people of nationalities within the bounds of Latin America.

NIH: National Institutes of Health; US Census: United States Census 2010.

The US government in 1997 replaced the ethnonym of Latino and Hispanic for the single term “Hispanic.”

### Geography and Latin American background

**Figure 1.**
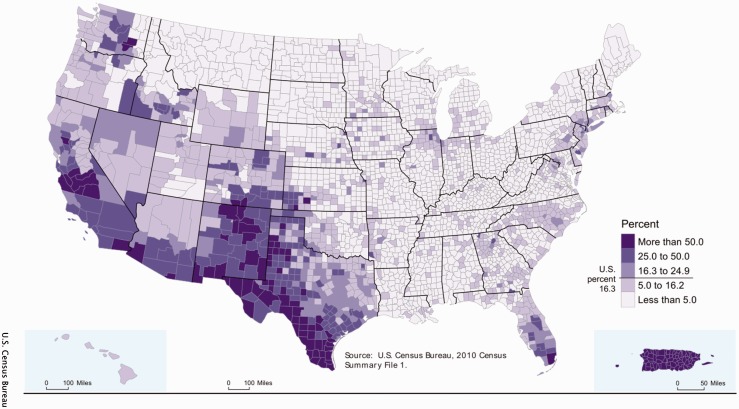
2010 United States (US) Census Hispanic population by county in the US.

In 2010, Hispanics comprised 16.3% of the total population of the US. Geographically, most reside in the southern regions of the West coast, which includes California and Texas and are characterized by a Mexican background (10% of the total population; 63% of the 2010 US Census self-identified “Hispanic or Latino” population) while the southern State of Florida and the Northeast of the US are predominantly of Puerto Rican, Cuban, and South American background (Figure 1; web 1). Hispanics remain the largest-growing minority in the US. Each Hispanic subpopulation is racially, socially and economically diverse. Compared with non-Hispanic whites, Hispanics in the US are more likely to live in poverty (21.4% vs. 11.6%) and less likely to have health insurance (web 2). These socioeconomic and geographical differences are considered important determinants of health and could potentially contribute to health disparities in MS.

### Genetics of Hispanic Americans

The US Hispanic population represents a complex and heterogeneous ethnic group with a genetic signature derived from three ancestral groups: the original indigenous inhabitants of the Americas, European settlers and to a lesser degree West Africans.^2,3^ The underlying genetic architecture of this population is primarily the result of two-way admixture between Native American and European populations or three-way admixture among Native American, European, and West African populations. Historical contributions from these three ancestral groups to the contemporary genetic structure of the US Hispanic population can differ geographically.^4^ Although all subpopulations contain some fraction of European ancestry, subgroups in the Northeast and Southeast US contain more West African ancestry than Hispanic subgroups from the Southwest US, which possess more Native American ancestry.^5^ Superimposed upon differences in admixture proportions among contemporary Hispanic populations, the ancestral populations themselves differ in their degree of genetic divergence; genetic differentiation between European and Native American populations appears to be greater than between Europeans and Africans.^6^

Over the years, the ability to disaggregate individuals using so-called ancestry informative markers (AIMs) has been a useful tool to help researchers account for ancestral variation in genetic studies of complex diseases. This process has been successfully applied in studies of Hispanic individuals affected with complex common diseases such as lupus, diabetes,^7^ and asthma.^8,9^ A study involving 884 Hispanic Americans with systemic lupus identified them to mainly consist of three groups: European (48%), Native American (40%), and West African (8%) background in differing individual proportions.^10^ A significantly higher risk of lupus was identified in those Hispanics with Native American ancestry. Similarly a study that evaluated asthma in Hispanics further underscores subgroup genetic variations in ancestral proportions between Hispanic Mexicans and Puerto Ricans.^11^ This known genetic diversity in Hispanics has the potential to affect the risk of MS, its clinical expression, and long-term outcomes.

## Epidemiology of MS in Hispanics

### Incidence, prevalence and mortality

MS as estimated by the World Health Organization shows a global median prevalence of 35 cases and a median incidence of 4 cases per 100,000, with a current total estimate of 2.3 million individuals affected with MS.^12^ There are important geographical differences with variable estimates across the world, including Latin America.^13^ In the US, the prevalence of 400,000 is indicative of all cases, including non-Hispanic whites, Hispanics, blacks and other minorities such as Asian and thus it is difficult to ascertain prevalence by race/ethnicity because of MS not being a reportable chronic disease such as on the island of Puerto Rico.^14^ Nevertheless, there have been a number of small population-based studies, including one conducted in 19 Texan counties, indicating a low prevalence of MS in Hispanics (11 per 100,000) compared to non-Hispanic whites (56/100,000) and non-Hispanic blacks (22/100,000).^15^ In 2003, an update to this study was conducted and again reported a higher prevalence of MS in non-Hispanic whites.^16^

In addition, in the last several years, published incident reports from two large multi-ethnic cohorts indicate that Hispanics in the US are reported to develop MS less frequently than non-Hispanic whites. A retrospective cohort study from the Kaiser Permanente plan in Southern California reported an incidence of 2.9 per 100,000 vs. 6.9 in whites.^17^ A second study using the US military-veteran population found that the incidence was still low for Hispanics when compared to whites and blacks. The estimated annual age-specific incidence rate for Hispanic was reported at 8.2%, significantly much lower than whites (9.3) and blacks (12.1).^18^ While limited, these two studies further highlight that race and ethnicity play a large role in the distribution of MS worldwide. The continued observation that Hispanics are affected by MS at a lower rate is both of interest and importance as research in this population progresses.

A continued marked variability in mortality measures in the US related to MS by race and ethnicity is also seen. Differences in Hispanic classification have been used. One of the earlier studies used Spanish surnames as a proxy for “Hispanic” and reported age-adjusted MS death rates of 0.27 between 1966 and 1975 compared to death rates of 0.83 in whites.^19^ Stratified results by place of birth were also provided, reporting Hispanic immigrant mortality to be extremely low at 0.07. Trends in MS mortality rates continue to support that white non-Hispanics are at increased risk compared to Hispanics.^20^ These continued observations of lower mortality rates and modification by place of birth in MS should present the opportunity to investigate if survival advantages exist in Hispanics and/or immigrants more generally. However, evidence for or against a Hispanic^21,22^ or immigrant^23^ paradox in MS is not yet available.

## Demographic features

The female-to-male sex ratio in Hispanics with MS is predominantly female with an average of 2:1.^24,25^ This is similar to reports of non-Hispanic whites. Differences however have been noted when stratifying by place of birth where female predominance is increased in those who have immigrated to the US.^26^ This difference is speculated to reflect migratory patterns in time where males make up a larger proportion.^27^

Several studies indicate that age of symptom onset or diagnosis is younger compared to whites^17,24,25,28,29^ across different regions in the US (Table 2(a)) and that the younger age of onset appears to be more pronounced in those who are US born (Table 2(b)).^25,30^ A study of 125 Hispanics with MS compared to 100 non-Hispanic whites reported that Hispanics developed their first symptoms earlier (28.4 ± 0.97 years) in contrast to whites (32.5 ± 1.37 years). The difference was significantly younger (*p* > 0.001) for those who were US born compared to immigrant Hispanics to the US at adolescent age or older with 74% developing MS in the US. An incident and population-based study from the Kaiser Permanente Group of MS diagnosis in whites, African Americans, and Hispanics observed significant differences with Hispanics being younger at diagnosis.^17^ This observation continues to be consistent even across other regions of the US. In a study where predominantly Hispanics of Caribbean background were included, Hispanics compared to whites had younger age at diagnosis and age at exam.^25^ However, although age at first symptom did not differ significantly between the two groups, the US born were significantly younger than their immigrant counterparts (Tables 2(a) and (b); *p* > 0.0001). The apparent differences in the age at first symptom between studies from different US regions could reflect cultural differences in utilization and access to care, socioeconomic status, and/or environmental and genetic admixture differences in these subpopulations. More recently, a study of Hispanics in the Eastern part of the US suggests that the age of onset for Hispanics is not only younger compared to whites but also compared to African Americans (Table 2(a)).^31^ Interestingly, large proportions of pediatric-onset patients who self-identified as Hispanic are also reported among pediatric cohorts of MS, which may reflect that ancestry as well as place of birth has the potential to increase susceptibility to early-onset MS.^32,33^

**Table 2.** (a) Multiple sclerosis clinical characteristics in Hispanics compared to whites by region in the continental United States (US).

	South East Coast^25^		West Coast^24^		West Coast^17^	
	Hispanic, *n* = 312	White, *n* = 312	Hispanic, *n* = 119	White, *n* = 76	Hispanic, *n* = 116	White, *n* = 258
First symptom, mean age	33.6	35.2	28.5	32.6	33.2	40.7
Diagnosis, mean age	38.1	40.9	29.7	32.9	35.1	44.5
Diagnosis lag, years	4.4	5.6	1.2	0.3		
Disease duration, years	12.4	16.7	8.8	11.4	10.7	13.5
Gender, female (%)	80	77	58	75	78	71
Initial presentation, percentage						
Sensory	47	49	13.9	27.9	n/a	n/a
Optic neuritis^a^	<25	<25	31.5	19.7	n/a	n/a
Spinal cord^b^	∼35	∼30	25.1	13.1	n/a	n/a

a Optic neuritis estimate for South East Coast was not specifically reported in the original reporting but extrapolated from a bar figure.

b Spinal cord estimate for South East Coast was not specifically reported in the original reporting but extrapolated from a bar figure.

**Table:** 

(b) Multiple sclerosis disease onset in US-born Hispanics compared to Hispanic immigrants to the US.				
	South East Coast^25^		West Coast^30^	
	US-born, *n* = 105	Immigrant, *n* = 207	US-born, *n* = 202	Immigrant (late), *n* = 67
First symptom, mean age	29.5	35.7	28.5	34.2
Diagnosis, mean age	32.5	41	30.1	36.6

## Clinical presentation

Cross-sectional studies have been informative in delineating clinical differences between Hispanic and non-Hispanic white populations despite the scarcity of reports that have examined such differences in MS.^1^ Several clinical characteristics of MS in Hispanics appear to differ from non-Hispanics whites. For example, several studies report variability in the frequency of initial MS symptoms. In an analysis of 468 individuals with clinical isolated syndrome, the first event to precede MS, 54% of Hispanics presented with optic neuritis, which is significantly higher than the 43% of whites presenting with optic neuritis (*p* = 0.006).^34^ Optic neuritis was also found to represent 30% of cases among a Hispanic pediatric MS cohort^35^ while the frequency of optic neuritis among the adult cases was 35%. Interestingly, a recent study that focuses on Hispanics of Caribbean background in the US reported sensory symptoms to be the most common presentation.^25^ These noted clinical discrepancies between different Hispanic subgroups may emanate from regional differences in environmental exposures or known ancestral genetic heterogeneity.

There are few data regarding the imaging characteristics observed in Hispanics. Nevertheless, a high proportion of complete transverse myelitis at presentation (25% compared to 13% whites) of disease has been reported mostly in Hispanics presenting with MS as adults on the West Coast.^35^ Interestingly, Amezcua et al. reported that more than 75% had cervical spinal cord lesions on magnetic resonance imaging (MRI) with a median time of diagnosis of two years at the time MRI was collected.^36^ In addition, about 20% were found to have the presence of longitudinal extensive spinal cord lesions (LESCLs). Because LESCLs are a common feature in neuromyelitis optica (NMO) spectrum disorders^37^ and other autoimmune conditions,^38^ NMO antibody was investigated as well as other autoimmune syndromes that could explain such findings and all were reported to be seronegative. The presence of LESCLs was seven times more likely to accompany ambulatory disability (odds ratio (OR) 7.3, 95% confidence interval (CI) 1.9–26.5; *p* = 0.003) compared to absence of spinal cord involvement. These results mirror Asian reports (14%–31% with LESCLs)^39–41^ and are in contrast to that reported in whites (1%–3%).^42,43^ Because a genetic link between Asians and Native Americans is supported by the sharing of particular human leukocyte antigen (HLA) alleles and haplotypes^44^ and Hispanics in the western US are primarily an admixture of Native American and European^45^ ancestries, Amezcua et al investigated global individual admixture and spinal cord involvement in 46 individuals with MS who self-reported Hispanic background.^1^ Using ancestral informative markers to assess genetic global ancestry, an increasing proportion of non-European ancestry was associated with an increased risk of LESCLs (*p* = 0.03) in addition to greater disability (*p* = 0.05). This relationship provides compelling evidence supporting the hypothesis: that shared clinical characteristics, such as patterns of spinal cord involvement, between Asian and Hispanic MS patients could derive from shared genetic traits.

### Disability

Disease progression following the diagnosis of MS has been reported to be similar between US Hispanics and white Americans.^17,24,25^ Interestingly, once stratified by MS subtype a larger proportion of Hispanics than whites with ambulatory disability were observed in those with relapsing MS.^25^ Nevertheless, an observation that most Hispanics present with relapsing disease and are younger at start of disease compared to white non-Hispanics may be confounding these observations.^25,29,46^ A recent study by Ventura et al. that used the Patient-Derived Multiple Sclerosis Severity Score (P-MSSS) reported significantly higher disability scores for Hispanics (3.9 ± 2.6) compared to whites (3.4 ± 2.6; *p* < 0.0001; adjusted for age).^29^

## Potential risk factors in Hispanics with MS

### Genetics

The involvement of genetic variation in MS has been well documented by numerous sibling risk, adoption, and twin studies.^47–50^ The clinical heterogeneity and complex etiology of MS have long been confounding factors for genetic studies of the disease. While the first confirmed MS genetic association with HLA allelic variants was identified in the early 1970s, further robust gene discoveries were limited until late 2007.^51^ Despite the technological and statistical advances in the study of complex genetic disease, we have uncovered only a small proportion of the genetic influences in MS. Much is yet to be understood regarding the HLA and the more than 110 additional susceptibility variants now identified in MS.^52^ We must explore how these known genetic factors influence not only disease susceptibility, but disease outcomes, therapeutics, and responses to environmental exposures. While our current research findings are revealing the genetic underpinnings of MS in individuals of Northern European descent, large genetic studies of MS in other populations have yet to be realized. The generalization of current findings to individuals of different genetic ancestry is a significant question to address, especially in light of reported differences in prevalence, clinical course, and progression of MS across populations.

As with many complex diseases, there is a paucity in genetic research conducted on multi-ethnic populations;^53^ the genetics of MS is no exception. While relatively less is known about the prevalence of MS in Latin America, available epidemiological surveys suggest that prevalence rates may be higher than once believed in Hispanics and their geographical regions.^1,54^ There are several factors that complicate the genetic study of MS in this population including the lack of consistent diagnostic criteria, greater awareness of the disease, increased prevalence of infectious disease, and ethnic heterogeneity.^55^ Despite progress (again almost exclusively in non-Hispanic populations) toward understanding the genetics of MS, the genes identified thus far represent only a fraction of the inherited susceptibility.^52^ While these findings demonstrate significant progress in identifying meaningful MS risk variation, the clinical variability of MS is likely to have roots in a complex genetic architecture that is heterogeneous across populations.

To date, genetic studies of MS have focused almost exclusively on the interrogation of genetic susceptibility in ancestral European datasets. There is much less known about the genetics of MS in Hispanics.^56–61^ Initial efforts to more broadly explore genetic variation suggest that individuals (*n* = 29) with MS and of Mexican background have a higher ancestral proportion of European ancestry compared to Mexican controls, with a notable enrichment across chromosome 6 (genetic location of the major histocompatibility complex (MHC) region).^62^

The underlying ancestral genetic differences between Hispanics and non-Hispanics may suggest that not all previously identified MS genetic susceptibility loci are relevant in the Hispanic population or that different risk haplotypes may exist across populations. Characterizing the genetic landscape of Hispanic MS patients is an active area of research that is beginning to gain traction.

### Environmental factors

As a complex disease, environmental factors are likely to play an important role in the development of MS. Sun exposure,^63^ vitamin D deficiency,^64^ cigarette smoking^65^ and Epstein-Barr virus^66^ are among the most widely studied. However, few studies have examined these factors in US Hispanics.

Overall, Hispanics in the US have lower vitamin D levels compared to whites as reported by the National Health and Nutrition Examination Survey.^67^ In MS, low levels of vitamin D have been linked to MS susceptibility only in whites, while no significant association has been found in Hispanics.^64^ Amezcua et al. compared vitamin D levels of Hispanics to those of white background with MS and found significant vitamin D deficiency in Hispanics (25.1 ± 9.4 ng/ml) with MS irrespective of season compared to whites (37.3 ± 19.8 ng/ml, *p* < 0.001).^68^ This study also examined physical disability in relationship to vitamin D status and while a relationship between higher disability and low vitamin D levels has been reported in whites, this study was not able to confirm a similar pattern in Hispanics. Hence, a relationship between vitamin D and risk of MS in Hispanics has not yet been confirmed despite observing a trend of lower levels. A large case-control study that examines vitamin D in Hispanic ethnicity and the risk of MS is currently underway.^69^

### Comorbidities

There is an increasing recognition that MS outcomes are influenced by multiple factors such as genetics and environment, but also comorbidity.^70^ The most common MS-reported comorbidities, including mental disorders such as depression, cardiovascular conditions (i.e. hypertension and hyperlipidemia), and respiratory conditions, have been described in primarily white prevalent cohorts.^71^ Mexican Americans are disproportionately afflicted by obesity and Type 2 diabetes and are often observed more often than higher acculturated Hispanics paralleling their age of immigration or time in the US.^72–75^ Comorbidities and their impact on Hispanics with MS in the US are limited. A study that surveyed 99 Hispanics in the US reported that almost 4 in 10 reported symptoms associated with depression with a significant impact on quality of life.^76,77^ In a study of individuals of primarily Mexican American background, 30% of the cohort (*n* = 304) was reported to have a comorbid condition. Immigrants to the US with MS reported more vascular risk factors, with hypercholesterolemia being the most common reported comorbid medical problem affecting them compared to US-born individuals (*p* = 0.002).^26^ A cross-sectional assessment of headache as comorbidity in individuals affected by MS found that chronic migraine was significantly more common among Hispanics (82%) than whites (18%; *p* = 0.012) and was noted to have significant impact on their daily life activities.^78^ Differences in comorbid condition by immigration status in the US Hispanic MS population is expected to be the result of health care disparities that could reflect sociocultural and economic barriers that could have withstanding effects on disability.

Several lines of evidence suggest that obesity during adolescence may contribute to the risk of MS. A multi-ethnic case-control study that had a high proportion of individuals of Hispanic MS background (*n* = 75, with 52% Hispanic) found that the risk of pediatric MS was highest among moderately and extremely obese teenage girls.^79^ However, it did not specifically address if ethnicity played a role in these findings. Given that the epidemic of obesity in the US continues with Hispanics being the second ethnic group most affected (adult: 42.5%; children: 22.4%), it is possible that it could affect the future incidence of MS and MS outcomes in this population. Interestingly, Hispanic ethnicity and obesity are two independent risk factors for Type 2 diabetes, and Native American ancestry has been reported to increase the risk of diabetes in Hispanics,^80^ which suggests that genetic admixture could also play a role in explaining comorbid differences observed in MS.

Parasites and microbes have been important for adjusting and forming the human immune system.^81^ While not technically a comorbidity, parasitic infections can coexist and have been noted to ameliorate MS in predominantly white background.^82,83^ Studies that examine parasitic infection differences between immigrants to the US and US-born Hispanics in MS are lacking. Similarly, microbiome changes due to changing diets and behaviors as a consequence of assimilation and/or acculturation in MS are also needed.

### Acculturation and the Hispanic culture

Hispanics in the US represent a complex population of American and foreign-born immigrants and both migration patterns and age at migration are reported to primarily influence MS disease susceptibility^84–86^ and age of onset.^24,87^ In addition, a higher proportion of those affected with MS have been reported to have disease severity among non-European immigrants to Norway, France, and United Kingdom compared with native-born individuals, despite similar access to health care and biological marker profiles, suggesting a possible role for acculturation factors.^87–89^

Acculturation is recognized to play a major role in the modification of social, behavioral, and health characteristics of immigrants, particularly of Hispanic immigrant groups in the US.^90^ The process refers to the cultural modification of an individual, group, or population by adapting to or borrowing traits from another culture after they migrate and can lead to a decline in health and mortality advantage over time.^90–93^ Using age of immigration and place of birth as proxy to acculturation, Amezcua et al. reported that late-immigrants (individuals age 15 years or older at time of immigration) were older at symptom onset (mean 34.2 ± 11.9 vs. 31.9 ± 12.9 vs. 28.5 ± 9.7 years, *p* < 0.001) compared to early immigrants (individuals immigrating to the US before age 15), with US born being significantly younger (Table 2(b)).^26^ In addition, a greater proportion of them had severe ambulatory disability (28% vs. 9% vs. 18%, *p* = 0.04) compared to early-immigrant and US born, respectively. There was no difference between groups by sex or type of MS and disease duration but differences were noted by socioeconomic status where the late immigrant was more commonly under the care of a public facility. In addition, older age of immigration to the US was found to be independently associated with increased ambulatory disability (adjusted OR 2.3, 95% CI 1.07–4.82; *p* = 0.03). Interestingly, the age of onset for the immigrant was well above the age of immigration to the US. Recognizing related social mechanisms such as acculturation, which could account for some of the variation seen in MS severity and related disability progression, is important (Table 3). Further studies that examine acculturation in the clinical care and self-management of MS in Hispanics are underway.

**Table 3.** Potential challenges in the treatments and care of multiple sclerosis (MS) in Hispanics in the United States (US).

Population changes in the US
Delayed diagnosis
Social, economic, and cultural factors influencing care
• Immigration
• Acculturation
• Perceptions
General lack of MS awareness in the community
Insufficient culturally relevant MS material and programs
Lack of cultural competent care by providers

Health disparities can also be rooted in cultural beliefs, which can lead to subsequent behaviors that can negatively affect health. Hispanic culture is characterized by strong values attached to family and cultural health beliefs.^94,95^ While limited, a qualitative study using focus group responses and direct inquiries (*n* = 106)^96^ participants reported MS to be caused by an environmental encounter such as stress (44%) with stress being significantly more common in individuals who were US born compared to immigrant Hispanics (81% vs. 60%, *p* = 0.01). Sociocultural factors identified included cultural idioms such as fright (*susto*), or sadness (*tristeza*), which significantly were more common in individuals who were immigrants compared to US born (30% vs 9%, *p* = 0.04). Interestingly, those who reported not being on disease-modifying treatments for MS were frequently those who believed MS to be the result of sociocultural factors. A study that assessed treatment adherence in pediatric-onset MS (*n* = 99; 47% Hispanics) reported 36% of Hispanics did not adhere to their disease-modifying treatment, with forgetfulness as the main cause.^97^ While we cannot speculate if cultural factors play a role in this decision, data from the focus group study suggest that Hispanics with MS draw on cultural belief systems in describing the causes of MS, which is consistent with other health literature in chronic medical conditions afflicting Hispanic populations.^98^ In addition, it highlights that these individuals may be taking rational actions that may be linked to non-adherence to treatment.^99^ Future studies are needed to better understand culture, perceptions, and attitudes in the relationship with MS and related disability.^100^ In addition, once identified, transforming perceptions and attitudes (i.e. taboos, values) into knowledge may prove to be a challenge.

## The development of a consortium

In spite of the shortage of MS studies in Hispanics within the US, several US-based cohorts have provided a large origin of data that are frequently mentioned in our review: (1) University of Southern California Hispanic MS Registry and (2) University of Miami Hispanic MS Registry are made up of incident and prevalent cases and are both actively recruiting new Hispanic participants. In order to maximize Hispanic diversity and deliver scalable results to this population, the first Hispanic MS network in the US was created in 2016: Alliance for Research in Hispanic MS (ARHMS.org). This consortium builds on existing biospecimen repositories with common clinical databases and is intended to be an active group of scientists and clinicians with broad representation across the US who care and treat Hispanics with MS. The overall goal of this group is to improve our understanding of this population epidemiologically, and investigate its genetic contributions to disease and sociocultural relationships in MS-related progression.

## Conclusion

We have reviewed the currently available data describing various aspects of MS in US Hispanics. While limited, it suggests that there are important clinical differences that are emerging such as a younger age of onset compared to whites, an influence of place of birth, and subpopulation differences as seen by differences in presentation. There are concerns that Hispanics, while potentially at less risk of developing MS, could be at higher risk of disability earlier in the course of disease. However, much more data are necessary to fully describe the clinical, genetic diversity, and environmental factors that might be involved. This includes a better understanding of the social, cultural, and economic barriers that Hispanics in the US are much more at risk of experiencing. Numerous studies are ongoing to help fill this gap in a comprehensive manner for the various Hispanic subgroups represented within the US and in collaboration with partners across the Americas.

## Appendix

Web 1: Ennis SR, Rios-Vargas M and Albert NG. The Hispanic population: 2010: 2010 Census brief. United States Census Bureau, C2010BR-04; May 2011, pp. 1–16, https://www.census.gov/prod/cen2010/briefs/c2010br-04.pdf (accessed 3 March 2017).

Web 2: Proctor BD, Semega JL and Kollar MA. Income and poverty in the United States: 2015, Report number: P60-256, https://www.census.gov/library/publications/2016/demo/p60-256.html (accessed 28 May 2017).
